# Mortality and years of life lost to death or disability by interpersonal violence against women in Brazil: Global Burden of Disease Study, 1990 and 2019

**DOI:** 10.1590/0037-8682-0287-2021

**Published:** 2022-01-28

**Authors:** Isabella Vitral Pinto, Nádia Machado de Vasconcelos, Rafael Bello Corassa, Mohsen Naghavi, Fatima Marinho, Deborah Carvalho Malta

**Affiliations:** 1 Universidade Federal de Minas Gerais, Programa de Pós-Graduação em Saúde Pública, Belo Horizonte, MG, Brasil.; 2 Fundação Oswaldo Cruz Minas, Instituto René Rachou, Belo Horizonte, MG, Brasil.; 3 Ministério da Saúde, Brasília, DF, Brasil.; 4 University of Washington, Institute for Health Metrics and Evaluation School of Medicine, Department of Health Metrics Sciences, Seattle, WA, United States of America.; 5 Vital Strategies, São Paulo, SP, Brasil.; 6 Universidade Federal de Minas Gerais, Escola de Enfermagem, Departamento de Enfermagem Materno Infantil e Saúde Pública, Belo Horizonte, MG, Brasil.

**Keywords:** Gender-based violence, Mortality, Injuries

## Abstract

**INTRODUCTION:**

Aggression against women is an important cause of morbidity and death. This study compares the variation of deaths and years of life lost to death or disability (DALY) caused by interpersonal violence against women in Brazil and its states.

**METHODS:**

This descriptive study analyzed estimates from the Global Burden of Disease Study (GBD) referring to interpersonal violence against women, aged 15 to 49 years, examining the mortality and DALY rates for Brazil and its states, in 1990 and 2019.

**RESULTS:**

In this study, 3,168 deaths of women between 15 and 49 years of age, caused by interpersonal violence, were estimated in 1990, and 4,262 in 2019, which represents an increase of 33.8%. Regardless of the Maria da Penha Law and the progress in policies for curbing violence against women, one can observe a stability in the mortality and DALY rates in most of the Brazilian states. Only Bahia had a significant increase in those rates, while Federal District, Rio de Janeiro, and São Paulo showed a significant decline.

**CONCLUSIONS:**

The rates of female homicide have remained stable when comparing 1990 and 2019. Although there were improvements in terms of women’s rights in the early 2000’s, the chauvinist and conservative society of Brazil has not been able to protect women, and the country might not reach the targets established by the UN’s 2030 Agenda.

## INTRODUCTION

Aggression is an important cause of death and of years of life lost to death or disability (DALY). Globally, 80% of all homicides are attributed to males, compared to only 20% to females^1^. However evidence shows that fatal violence against women is backgrounded by gender-based discrimination, often taking place in the context of intimate and affective relationships, and represents the closure of a continuum of violence and aggression, characterizing those deaths as “announced” or avoidable^2^ ^,^ ^3^ ^,^ ^4^ ^,^ ^5^. International literature indicates that there is a high risk of female homicide to be perpetrated by intimate partners and family members, between 38.6% and 48% of these events^1^ ^,^ ^6^ ^,^ ^7^.

In an attempt to qualify violent deaths of women, Brazil changed its Penal Code, creating the legal concept of femicide, understood as the homicide against women for reasons related to being female^8^. Such a definition covers cases of domestic and family violence, as well as the mistreatment of or discrimination against women^8^. Thus, femicide is part of the male domination and patriarchy, rooted in our society and culture, which could be considered the final step of chronic physical, emotional, or sexual aggressions^4^. In that sense, identifying femicides is essential to fighting impunity in those cases, breaking the notion of “normality”, which is historically and culturally attributed to such events. Moreover, it helps to demonstrate how gender-based power inequalities work to increase women’s vulnerability to these crimes, thus providing evidence to guide prevention policies^9^.

By using police data from states and crime reports, the Brazilian Forum of Public Security has monitored cases of femicide reported by authorities since 2016, the first year in which the law took effect. The researchers observed an increase of 43% in the reports of femicide, from 929 events in 2016 to 1,326 events in 2019^10^. Since identification, recording, and investigation require the training of public security professionals, as well as structural and personnel conditions, it is questionable whether this increase shows an improvement in reporting or a real increase in the number of cases^10^.

Considering recent femicide registration by police and the limitations in identifying those cases in the Declarations of Death (DD), because there is no record of the circumstances of the crime, the analysis of data on female homicides may contribute as a *proxy* to understand the evolution of the problem in this population. The main source of data about mortality in Brazil are the DD, which comprise the Mortality Information System (SIM, in Portuguese) and are also the standard document for data collection for epidemiological purposes and vital statistics^11^.

From the early 2000’s on, there has been an important investment in the improvement of the quality of information about deaths in SIM, and one can note an increase in the coverage of reports in Brazil, reaching 95%, as well as a reduction in the number of ill-defined causes of death^12^ ^,^ ^13^. However, there are still problems in the coverage of SIM in some regions, with the presence of ill-defined causes, deaths with undetermined intention, and incomplete diagnoses, such as homicides by unspecified means, which makes it more difficult to obtain a true diagnosis of these events^13^. To minimize this problem, several actions have been taken to estimate mortality rates more properly and to improve the quality of the data from SIM^12^ ^,^ ^13^. One of these strategies was to include the Ministry of Health in the network of the Global Burden of Disease Study (GBD), together with Universidade Federal de Minas Gerais and the Institute for Health Metrics and Evaluation (IHME) of the University of Washington in the United States of America^14^.

Since the 1990s, the GBD has advanced in terms of innovative methodologies to evaluate the loss of health caused by diseases, lesions, and risk factors, generating comparable estimates for several countries around the world^14^ ^,^ ^15^. In Brazil, the main source for GBD data on mortality is the SIM, which is corrected and adjusted with the use of other national and international sources^15^. In addition to estimating the absolute values and death rates, the GBD proposed the calculation of years of life lost to premature death or disability (DALY), a measurement which quantifies and classifies the burden of diseases due to specific causes, contributing to the understanding of its evolution over time^16^.

According to GBD data, in 2019, Brazil had the 25th highest death rate and 26th highest DALY rate by interpersonal violence against women when compared to all countries^17^. In that year, the number of deaths by interpersonal violence against girls and women in Brazil was 5.7 (95% uncertainty intervals (UI): 5.4; 6.1)/100,000, which reached a death rate of 5.2 (95% UI: 4.9; 5.5) /100,000, while the DALY rate was 317.4 (95% UI: 298.3; 338.8)/100,000, representing the 24th most important burden for women^17^. However, the problem seems to be more serious in the age group of 15 to 49 years, in which the number of deaths for interpersonal violence in 2019 was 4,240 (95% UI: 4.3; 4.5)/100,000, the death rate was 7.3 (95% UI: 6.8; 7.7)/100,000, and the DALY rate was of 472.1 (95% UI: 442.2; 503.8)/100,000, representing the ninth most important burden^17^.

The study of homicides and the loss of health caused by these events, as well as the use of comparable estimates corrected over the years by the GBD, may help to generate evidence on the evolution of female homicides and may aid in rethinking the policies adopted to tackle violence against women in Brazil. Therefore, this study aimed to compare the variation of deaths and DALYs caused by interpersonal violence against women in Brazil and its states, considering the years of 1990 and 2019.

## METHODS

This is a descriptive study based on the GBD 2019^18^ estimates of mortality and DALYs caused by interpersonal violence against women aged 15 to 49 years. The data presented in this study, such as numbers, rates, variations and uncertainty intervals were generated by GBD 2019^18^ and available for public access in the GBD Compare^17^ site.

The GBD organizes causes of death, disease, and lesions in four hierarchical levels. In the first, the causes are organized by: communicable, maternal, neonatal, and nutritional diseases; chronic non-communicable diseases; and lesions. Within the lesions, there are three causes in the second level: transportation accidents, unintentional lesions, self-inflicted lesions, and interpersonal lesions. In the third level of this last cause, there are self-inflicted lesions, interpersonal lesions, conflict and terrorism, as well as conflicts and homicides by police. The present study selected, out of this third level, only the “interpersonal violence” cause, corresponding to codes X85 to Y08.9 and Y87.1 of the International Disease Code - 10th Edition (ICD-10). The 4th level includes physical violence by firearms (X93 to X95.9), physical violence by sharp or pointed object (X99 to X99.9), physical violence by other means (X85-X92,9; X96-X98.9; Y00-Y04.9; Y06-Y08.9; and Y87.1), and sexual violence (Y05). Sexual violence is considered only as a cause of morbidity, as it is part of the DALY, but it is not included in the calculation of mortality by interpersonal violence^19^.

This article evaluated the death rate by interpersonal violence against women according to age groups and the death rate by interpersonal violence against women aged 15 to 49 years, for each year from 1990 to 2019. In addition, we analyzed the GBD estimates of mortality and DALY rates caused by interpersonal violence and its level four in Brazil, for 1990 and 2019. For the analysis of the 27 states, the death rate and the interpersonal violence DALY rate were considered. To evaluate if there was a statistically significant difference at the 5% level, the UI^20^ of the estimates were compared.

The GBD estimate process is based on the identification of multiple data sources, including census data, surveys, public records and vital statistics, administrative data from healthcare services, disease notification, among other sources^18^. This data is identified through a systematic review of published studies; surveys by the government and by international organizations; primary sources, such as Research in Health and Demographics; as well as data banks provided by a network of collaborators^18^. It is worth mentioning that the GBD formulates the correction of death data by redistributing ill-defined or non-specific causes of death, such as events of undetermined intent^18^. The calculation of the DALY considers the years of life lost to premature death (Years of Life Lost - YLL) and the years of life lost to disability caused by disease, after-effects, or deficiency (Years Lived with Disability - YLD)^15^.

For this study, we selected female individuals, aged 15 to 49 years. Since there are differences in the age structure of the 27 states of Brazil, the standardized rates of interpersonal violence against women were calculated to verify if there were differences in comparison to the death rates in the GBD Compare. As a standard, we took the age structure used by the GBD and the population estimated by the GBD for each state in the evaluated years. The standardized mortality rates for the population of women, aged 15 to 49 years, for the states in 1990 and 2019, were similar to those in the GBD Compare. Therefore, the results shown considered the non-standardized rates of women, aged 15 to 49 years (Supplementary Material).

The Global Burden of Disease Study in Brazil (GBD Brazil) was approved by the Committee of Ethics in Research at the Federal University of Minas Gerais (Project CAAE - 62803316.7.00005149).

## RESULTS

**FIGURE 1:**
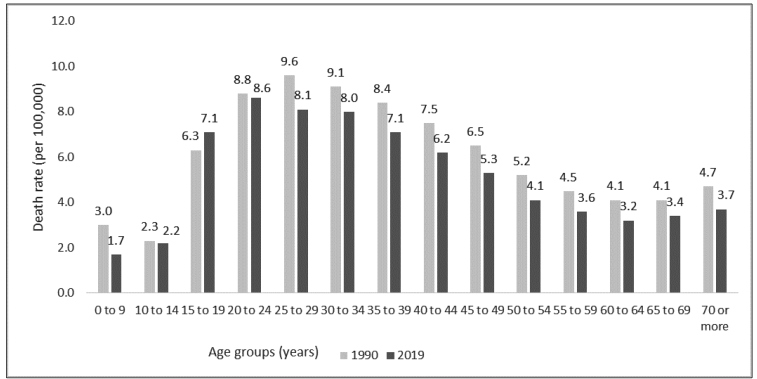
Death rate for interpersonal violence against females, according to age groups. Brazil, 1990 and 2019.

The death rate by interpersonal violence against girls and women of all ages in Brazil has changed from 5.8 (95% UI: 5.6; 6.0)/100,000 in 1990 (ranking 20th place among all causes of death) to 5.2 (95% UI: 4.9; 5.5)/100,000 in 2019 (ranking 25th place among all causes of death), but there was no statistically significant difference between the two years (data not shown in the tables and figures). Analyses by age group indicated that young and adult women, aged 15 to 49 years, were at a higher risk of dying from interpersonal violence, compared to other age groups (Figure 1). The highest death rate by interpersonal violence in 1990 was found for women of 25 to 29 years of age (9.64/100,000), while in 2019, the highest death rate was for even younger women, 20 to 24 years of age (8.58/100,000) (Figure 1).

**Source:**Institute for Health Metrics and Evaluation. Global Burden of Disease Study 2019.

The evaluation of the death rate by interpersonal violence for women 15 to 49 years of age, for each year, shows that it remained relatively stable. Considering the year by year evaluation, a significant increase was only found between 1992 and 1993, and between 1994 and 1995. Using 2006 as a reference (the year when Maria da Penha Law was passed), a significant difference can only be observed in 2019 (Table 1).

**TABLE 1:** Death rate for interpersonal violence against women, aged 15 to 49 years, according to the year of death. Brazil, 1990 to 2019.

Year	Death rate	Uncertainty Interval	Year	Death rate	Uncertainty Interval
	(per 100,000)	(95%)		(per 100,000)	(95%)
1990	8.1	(7.9;8.4)	2006*	8.0	(7.8;8.2)
1991	8.2	(7.9;8.5)	2007	7.7	(7.5;7.9)
1992	7.8	(7.6;8.1)	2008	7.7	(7.5;8.0)
1993	8.5	(8.2;8.7)	2009	7.9	(7.7;8.1)
1994	8.8	(8.5;9.0)	2010	8.0	(7.8;8.3)
1995	9.6	(9.3;9.9)	2011	7.9	(7.7;8.2)
1996	9.4	(9.2;9.7)	2012	8.0	(7.8;8.3)
1997	9.2	(9.0;9.4)	2013	7.9	(7.7;8.2)
1998	9.1	(8.9;9.3)	2014	8.0	(7.7;8.2)
1999	8.7	(8.5;8.9)	2015**	7.7	(7.5;8.0)
2000	8.8	(8.6;9.1)	2016	7.7	(7.4;7.9)
2001	8.8	(8.6;9.0)	2017	7.8	(7.5;8.0)
2002	8.7	(8.5;9.0)	2018	7.5	(7.1;7.8)
2003	8.5	(8.3;8.7)	2019	7.3	(6.8;7.7)
2004	8.3	(8.1;8.6)			
2005	8.1	(7.9;8.4)			

**Observations:** *Year of publication of Law 11,340/2006, better known as the Maria da Penha Law^21^; **Year of publication of Law 13,104/2015^8^.

**Source:** Institute for Health Metrics and Evaluation. Global Burden of Disease Study 2019.

In 1990, 3,168 deaths were estimated for women of 15 to 49 years of age by interpersonal violence, while in 2019, 4,262 deaths, which reveals a significant increase of 33.8%. An important proportion of these events was caused by firearms, reaching 55.6% in 2019. The number of deaths by physical violence by firearms and the number by sharp, pointed objects showed significant increases between the evaluated years, 69.6% and 50.1%, respectively. Regarding the number of deaths by physical violence by other means, there was a significant decline of 14.8% (Table 2).

**TABLE 2:** Number of deaths, death rate, and DALY rate for interpersonal violence against women, aged 15 to 49 years, and percentage variation of the measurements with 95% UI. Brazil, 1990 and 2019.

Measurement	Cause	1990	2019	Variation 1990-2019
		(95%UI)	(95%UI)	(95% UI)
Number of Deaths	Interpersonal violence	3.168 (3.057;3.277)	4.240 (4.003;4.515)	33.9 (25.8;45.5)
	Physical violence by firearm	1.495 (1.436;1.556)	2.356 (2.214;2.510)	57.6 (46.7;71.7)
	Physical violence by sharp object	708 (680;739)	1.063 (999;1.136)	50.1 (39.7;62.8)
	Physical violence by other means	965 (926;1.006)	822 (767;892)	-14.8(-21.1;-6.4)
				
Death Rate (per 100,000)	Interpersonal violence	8.1 (7.9;8.4)	7.3 (6.8;7.7)	-10.9 (-16.3;-3.1)
	Physical violence by firearm	3.8 (3.7;4.0)	4.0 (3.8;4.3)	4.9 (-2.4;14.3)
	Physical violence by sharp object	1.8 (1.7;1.9)	1.8 (1.7;1.9)	-0.1 (-7.0;8.4)
	Physical violence by other means	2.5 (2.4;2.6)	1.4 (1.3;1.5)	-43.3 (-47.5;-37.7)
				
DALY Rate (per 100,000)	Interpersonal violence	542.8 (518.1;568.0)	472.1 (442.1;503.8)	-13.0 (-17.8;-6.4)
	Physical violence by firearm	232.3 (223.2;242.2)	239.9 (225.6;255.4)	3.3 (-3.9;12.4)
	Physical violence by sharp object	110.6 (106.1;115.4)	105.0 (98.9;112.2)	-5.0 (-11.4;2.7)
	Physical violence by other means	159.2 (151.7;167.3)	86.4 (80.8;93.5)	-45.7 (-49.4;-40.9)
	Sexual violence	40.7 (26.1;58.4)	40.7 (26.1;58.3)	0.1 (-4.0;4.4)

**Source:** Institute for Health Metrics and Evaluation. Global Burden of Disease Study 2019.

The death rate by interpersonal violence against women of 15 to 49 years of age in 1990 was 8.1 (95% UI: 7.9; 8.4)/100,000, which is the 5th most important cause of death. Meanwhile, in 2019, the rate was 7.3 (95% UI: 6.8; 7.7)/100,000, reaching 3rd place. In other words, there was a statistically significant decline of 9.9%. By contrast, the death rate by firearms went from 3.8 (95% UI: 3.7; 4.0) /100,000 to 4.0 (95% UI: 3.8; 4.3)/100,000 in 2019, showing no difference in the period. The death rate by sharp, pointed objects also remained stable when the two years were compared, while the death rate caused by physical violence by other means showed a significant decrease of 44.0% (Table 2).

Finally, the DALY rate by interpersonal violence against women aged 15 to 49 years showed a decline of 13%, going from 542.8 (95% UI: 518.1; 568.0)/100,000 in 1990 (11th place in the ranking of causes of death with the highest burden), to 472.1 (95% UI: 442.1; 503.8)/100,000 in 2019, moving up to 9th place. The same pattern happened to the DALY rate of physical violence by other means, which decreased 45.7% between the evaluated years. No significant variation was found in the DALY rate of violence by firearms, or by sharp, pointed object, nor in the DALY rate of sexual violence (Table 2).

Comparisons between the years of 1990 and 2019, stratified by state, revealed non-significant changes in death rates and DALY rates in most states. Only the state of Bahia showed a significant increase in those rates, reaching a 77.2% increase in the death rate. On the other hand, three states showed a reduction in the two rates: the Federal District, Rio de Janeiro, and São Paulo. The negative variation of the death rate by interpersonal violence was 27.9%, 50.7%, and 47.6%, respectively (Table 3).

**TABLE 3:** Death and DALY rates caused by interpersonal violence against women, aged 15 to 49 years, percent variation of rates between 1990 and 2019 and 95% uncertainty intervals according to states. Brazil, 1990 and 2019.

State	Death Rate (per 100,000)		Variation of Death Rate	DALY rate (per 100,000)		Variation of DALY rate
	1990	2019	1990-2019	1990	2019	1990-2019
	(95% UI)	(95% UI)	(95% UI)	(95% UI)	(95% UI)	(95% UI)
Acre	9.0 (7.5;10.5)	7.4 (6.3;8.6)	-17.5(-33.1;1.6)	608.0 (512.3;703.8)	490.3 (424.0;560.7)	-19.3(-33.2;-1.9)
Alagoas	10.1 (8.6;11.6)	10.8 (9.0;13.1)	7.7(-16.3;40.6)	667.6 (576.4;757.1)	691.5 (581.2;824.2)	3.6(-17.5;31.5)
Amapá	6.6 (5.1;7.9)	7.6 (6.6;8.6)	14.9(-7.7;49.9)	467.7 (372.2;553.4)	507.3 (446.6;571.3)	8.5(-11.0;37.4)
Amazonas	6.6 (5.3;7.8)	6.8 (5.7;8.2)	3.5(-18.3;33.7)	458.6 (378.6;539.1)	456.7 (391.0;532.9)	-0.4(-19.2;25.8)
Bahia	5.7 (4.7;6.8)	10.1 (7.8;12.6)	76.8(31.3;139.2)	398.3 (337.8;464.5)	642.2 (509.3;798.0)	61.2(24.3;111.2)
Ceara	6.8 (5.4;8.4)	9.2 (7.1;11.9)	36.0(-1.8;89.5)	461.4 (376.9;559.7)	591.3 (463.7;747.9)	28.2(-4.3;71.8)
Distrito Federal	7.2 (6.3;8.2)	5.2 (4.4;6.2)	-27.0(-40.6;-8.2)	487.6 (433.5;551.5)	355.0 (305.2;419.2)	-27.2(-39.2;-10.9)
Espirito Santo	12.5 (11.6;13.4)	12.5 (10.3;14.8)	-0.1(-17.8;20.3)	808.9 (750.3;869.3)	775.1 (647.9;907.2)	-4.2(-19.7;14.3)
Goiás	12.1 (10.1;14.2)	10.9 (8.6;13.5)	-10.0(-31.1;18.1)	786.5 (670.3;907.7)	684.6 (555.6;841.3)	-13.0(-31.7;12.1)
Maranhão	7.3 (5.4;9.9)	6.7 (5.0;8.8)	-7.8(-38.0;35.8)	489.3 (376.3;645.2)	433.9 (330.5;548.9)	-11.3(-37.8;24.8)
Mato Grosso	7.4 (5.4;9.3)	8.5 (7.1;10.0)	13.9(-13.7;61.9)	503.8 (376.3;618.0)	539.2 (455.7;627.2)	7.0(-16.9;47.6)
Mato Grosso do Sul	9.6 (8.5;10.6)	7.6 (6.3;9.1)	-21.1(-35.5;-2.7)	630.3 (563.7;694.9)	491.2 (416.5;576.8)	-22.1(-34.6;-5.8)
Minas Gerais	5.6 (5.0;6.1)	6.6 (5.6;7.8)	19.2(-0.7;42.7)	380.2 (345.4;416.9)	430.8 (373.1;493.3)	13.3(-3.9;32.7)
Para	7.8 (6.3;9.3)	8.8 (7.4;10.5)	13.8(-10.2;48.5)	526.5 (433.4;618.9)	578.3 (491.1;676.4)	9.9(-11.5;39.1)
Paraíba	8.8 (7.6;10.1)	8.8 (7.2;10.6)	-0.7(-23.9;26.0)	587.1 (512.0;669.1)	567.4 (471.1;676.6)	-3.4(-23.5;18.8)
Paraná	5.9 (5.5;6.3)	6.9 (5.8;8.2)	17.4(-2.9;41.6)	401.1 (371.7;433.4)	450.0 (384.6;521.4)	12.2(-4.8;32.8)
Pernambuco	11,1 (10,1;12,0)	10,6 (8,7;12,5)	-5.0(-23.6;16.5)	720,9 (656,8;781,4)	668,6 (559,7;783,4)	-7.3(-24.2;12.1)
Piauí	4.5 (3.8;5.2)	4.6 (3.8;5.6)	2.9(-19.4;30.8)	319.9 (273.4;367.6)	319.3 (268.4;379.5)	-0.2(-18.6;22.3)
Rio de Janeiro	15.0 (14.1;15.9)	7.4 (6.2;8.7)	-50.6(-58.8;-41.4)	959.9 (898.9;1021.5)	480.5 (411.9;558.4)	-49.9(-57.2;-41.4)
Rio Grande do Norte	5.4 (4.5;6.5)	7.8 (6.1;9.8)	43.6(6.3;88.4)	376.4 (319.1;442.6)	512.2 (411.3;633.2)	36.1(4.8;71.9)
Rio Grande do Sul	6.2 (5.8;6.6)	6.9 (5.9;8.3)	11.7(-7.4;34.8)	423.6 (393.5;456.4)	450.6 (384.0;528.0)	6.4(-9.5;25.8)
Rondônia	11.0 (7.9;13.8)	9.6 (7.8;11.6)	-12.9(-36.0;29.6)	729.4 (528.1;901.5)	611.8 (514.2;722.8)	-16.1(-37.0;21.0)
Roraima	10.9 (7.8;13.5)	10.2 (8.8;11.5)	-7.0(-27.2;28.2)	717.2 (521.3;877.4)	661.7 (580.5;743.6)	-7.7(-26.9;24.7)
Santa Catarina	4.2 (3.8;4.6)	4.2 (3.5;5.0)	0.5(-18.3;24.2)	301.7 (272.0;334.1)	290.6 (247.6;340.2)	-3.7(-18.9;14.6)
São Paulo	8.2 (7.6;8.9)	4.3 (3.6;5.1)	-47.7(-56.9;-37.4)	548.0 (506.7;594.4)	294.9 (253.4;341.1)	-46.2(-54.2;-37.3)
Sergipe	8.1 (6.9;9.4)	8.5 (6.8;10.6)	5.6(-19.7;38.7)	541.5 (469.3;627.4)	545.7 (446.4;667.0)	0.8(-21.0;28.7)
Tocantins	7.4 (6.0;8.7)	7.3 (5.8;9.0)	-0.6(-23.3;28.9)	500.2 (416.4;585.2)	479.2 (389.8;578.1)	-4.2(-23.9;20.5)

**Source:** Institute for Health Metrics and Evaluation. Global Burden of Disease Study 2019.

## DISCUSSION

Analysis of the GBD data between 1990 and 2019 showed an increase in the absolute number of deaths by interpersonal violence, physical violence by firearms, and physical violence by sharp, pointed objects in Brazil. However, there was a reduction in the death rates by interpersonal violence against women over the 30-year period in Brazil. Concerning the DALY rates, a decline was observed in the burden of violence in this segment of the population, although it still ranks as the 9th cause of DALY. Assessment of the rates stratified by state demonstrated non-significant changes in most states.

Between 1990 and 2019, there were periods of more investment and development of public policies to reduce violence against women, as well as periods of stagnation and resistance to progress. The fact that Brazil is a signatory of the World Conference on Women, the 1979 Convention on the Elimination of all Forms of Discrimination Against Women (CEDAW), and the 1994 Inter-American Convention on the Prevention, Punishment, and Eradication of Violence Against Women (known as Convenção Belém do Pará), suggests that the country is committed to protecting and guaranteeing the rights of women, although this has been taking place at a slow pace. 

Some important advances towards giving more visibility to the women’s rights agenda include the creation of the Special Secretariat for Women's Policies (SPM), with the status of a Ministry, the holding of Conferences on Policies for Women in the three levels of government, the elaboration of the National Plan for Policies for Women, the implementation of the National Policy to Combat Violence Against Women^21^, as well as the enactment of Law 11,340/2006^22^ and Law 13,104/2015^8^. These documents also contributed to the development of strategies to fight against and prevent violence through intersectoral actions and articulation between different elements of the public society and the government. All this political and institutional context reinforced the need for creating specialized services to support women in a situation of violence in order to prevent femicide^23^.

However, since 2014, there has been an increase in conservative standings in Brazil, compromising democratic discussions and demands by the feminist movements in matters related to gender, sexual rights, and reproductive rights^23^ ^,^ ^24^. In 2016, after President Dilma Roussef was ousted, the SPM lost its Ministry status, which constituted the dismantling of a structure that was more open to demands for rights and that was essential in the fight to develop public policies for women^23^.

To this complex scenario, we must add the adoption of neoliberal policies, including budget freezes targeting public policies and the relaxation of gun control policies, leading to increased access to firearms^25^, measures that go against evidence in terms of preventing violence and promoting a healthy society^26^. Therefore, one can assume that the repercussions of the current setbacks will have a negative impact on women's life and health conditions in the short, middle, and long terms.

A study by the Institute of Applied Economic Research (IPEA) identified that the Maria da Penha Law generated significant effects in terms of reducing the homicides of women motivated by gender issues^27^. Likewise, the present study demonstrated a reduction in the death and DALY rates for interpersonal violence in Brazil. However, the evaluation according to states showed that such a significant decline occurred in only three states, while one showed a significant increase and the remaining states showed stability in the measurements during the 30-year period. We believe that the public policies for fighting violence against women have contributed to preventing this scenario from worsening.

Nevertheless, the impact of public policies addressing violence against women in Brazil has not reached all social groups equally. Analyses based on data from the SIM, from 2000 to 2017, have shown that death rates by homicide among young white and black women have differed substantially. Homicide rates among black women were nearly twice that observed for white women, with an increasing trend of 2.1% per year, while homicide rates among white women decreased by 0.8% per year^28^.

The literature consistently demonstrates that racial inequalities in the macrosocial context of Brazil produce a social hierarchy marked by disadvantages among black individuals, especially among black women^29^ ^,^ ^30^.

Research has shown that there is a connection between female homicides and places with high rates of male homicides, large urban areas or more densely populated places, income inequality, involvement with organized crime, drug trafficking, and scenarios in which the patriarchal structure is more rigid^2^ ^,^ ^3^ ^,^ ^4^. This fact shows a close relation between femicide and socioeconomic inequalities, and reinforces the need for intersectoral work and more permanent public policies to solve the problem^31^. 

The state of Bahia, which had a considerable increase in death and DALY rates from interpersonal violence, showed increases in general homicide rates in the period of 2008 to 2018^25^. Contrarily, the Federal District and the state of São Paulo, which showed a decline in death and DALY rates, also showed decreasing trends in general homicide rates between 2008 and 2018^25^. Moreover, when the time trends of homicides in the country is analyzed, one must consider the following: demographic changes in an aging population; the effect of the Disarmament Statute, which played a key role in curbing the increase in numbers of fatal violence; the state’s public security policies on crime prevention and control; the wars and armistices between organized crime groups; and the changes in the data quality from SIM, which demonstrated a considerable increase in violent deaths of undetermined intent from 2018 onwards^25^. Therefore, we believe that all these factors impacted the dynamics of female homicides.

The importance of monitoring female homicides and femicides was established by the definition of specific indicators in the United Nations (UN) 2030 Agenda^32^, in which the 193 UN members made a commitment to eliminate gender-based violence in public and private spheres^31^. In Brazil, the proposed goal for Objective 16 consisted of “a significant reduction in all forms of violence and the mortality rates related to it, in all places, including a reduction of ⅓ in the rates of femicides, homicides of children, adolescents, young adults, blacks, women, indigenous people, and LGBT populations”^33^. However, considering the profile of the homicides of women aged 15 to 49 years in Brazil, from 1990 to 2019, that 2030 goal may not be achieved.

One limitation of the GBD data that must be considered is that it does not stratify data according to race/color and to the location where the homicide occurred. The Violence Atlas of 2020^25^ showed that 68% of the women murdered in Brazil in 2018 were black. More alarmingly, this document demonstrates that, in 2017 and 2018, the rate of homicides for black women increased by 12.4%, while the rate for non-black women witnessed a decline of 11.7%^25^. Furthermore, researchers have used homicide in the home as a proxy to calculate the occurrence of femicide^25^. In 2018, deaths at home corresponded to 30.4% of all homicides, an increase of 6.6% in comparison to 2017^25^. Additionally, the Year Book published by the Brazilian Public Security Forum in 2020 showed that most femicides (58.9%) occurred at home^10^.

Death rates from interpersonal violence against women aged 15 to 49 years remained stable in most of the Brazilian states, when comparing the years of 1990 and 2019, regardless of the Maria da Penha Law and the improvements in the women's rights policies and of the fight against violence developed in the early 2000’s. Only three states managed to significantly reduce the death and DALY rates for interpersonal violence. 

Illustrating this scenario with reliable and comparable data is important to provide evidence to policymakers that can guide the design of more effective and equitable public policies. There is a strong concern that loosening gun control regulations, combined with the spread of a conservative, chauvinistic, and misogynist mentality will worsen the current scenario. Considering that, there is a risk that the country will not reach the goals for the 2030 Agenda for Sustainable Development. Tackling violence and femicide requires coordinated, intersectoral actions, involving governmental and non-governmental institutions and society as a whole in order to ensure that women’s rights are protected.
