# Supplementary material for: Mortality and years of life lost to death or disability by interpersonal violence against women in Brazil: Global Burden of Disease Study, 1990 and 2019
Source: Rev Soc Bras Med Trop. 2022 Jan 28;55(Suppl 1):e0287-2021. doi: 10.1590/0037-8682-0287-2021 (PMC9009422; doi:10.1590/0037-8682-0287-2021)
Supplement: Supplementary file 1 [file 1678-9849-rsbmt-55-s01-e0287-2021-supp1.pdf]

Supplementary Table: Death rate and death rate standardized caused by interpersonal violence against women, aged 15 to 49 years, according to states. Brazil, 1990 and 2019.

Location	Year	Death rate (per 100,000)	Death rate standardized* (per 100,000)
Acre	1990	9,0	8,8
Alagoas	1990	10,1	10,1
Amapá	1990	6,6	6,4
Amazonas	1990	6,6	6,5
Bahia	1990	5,7	5,8
Ceará	1990	6,8	6,8
Distrito Federal	1990	7,2	7,1
Espírito Santo	1990	12,5	12,4
Goiás	1990	12,1	11,9
Maranhão	1990	7,3	7,6
Mato Grosso	1990	7,4	7,5
Mato Grosso do Sul	1990	9,6	9,5
Minas Gerais	1990	5,6	5,6
Pará	1990	7,8	7,8
Paraíba	1990	8,8	8,9
Paraná	1990	5,9	5,9
Pernambuco	1990	11,1	11,3
Piauí	1990	4,5	4,6
Rio de Janeiro	1990	15,0	14,8
Rio Grande do Norte	1990	5,4	5,5
Rio Grande do Sul	1990	6,2	6,2
Rondônia	1990	11,0	11,0
Roraima	1990	10,9	10,8
Santa Catarina	1990	4,2	4,2
São Paulo	1990	8,2	8,1
Sergipe	1990	8,1	8,3
Tocantins	1990	7,4	7,4
Acre	2019	7,4	7,4
Alagoas	2019	10,8	10,9
Amapá	2019	7,6	7,5
Amazonas	2019	6,8	6,8
Bahia	2019	10,1	10,1
Ceará	2019	9,2	9,2
Distrito Federal	2019	5,2	5,3
Espírito Santo	2019	12,5	12,6
Goiás	2019	10,9	10,9
Maranhão	2019	6,7	6,7
Mato Grosso	2019	8,5	8,5
Mato Grosso do Sul	2019	7,6	7,6
Minas Gerais	2019	6,6	6,6
Pará	2019	8,8	8,8

Paraíba	2019	8,8	8,9
Paraná	2019	6,9	7,0
Pernambuco	2019	10,6	10,7
Piauí	2019	4,6	4,6
Rio de Janeiro	2019	7,4	7,6
Rio Grande do Norte	2019	7,8	7,9
Rio Grande do Sul	2019	6,9	7,0
Rondônia	2019	9,6	9,6
Roraima	2019	10,2	10,1
Santa Catarina	2019	4,2	4,2
São Paulo	2019	4,3	4,3
Sergipe	2019	8,5	8,5
Tocantins	2019	7,3	7,3

Observations: *According to Global Burden of Diseases world population age standard.

Source: Institute for Health Metrics and Evaluation. Global Burden of Disease Study 2019.
